# Assessment of Knowledge, Attitude, and Practices (KAP) Toward Hepatitis B Infection, Its Prevention, and Vaccination Among Health Care Workers

**DOI:** 10.7759/cureus.39747

**Published:** 2023-05-30

**Authors:** Manjri Garg, Banoth Sridhar, Virender Katyal, Sandeep Goyal

**Affiliations:** 1 Medicine, Pandit Bhagwat Dayal Sharma Post Graduate Institute of Medical Sciences, Rohtak, IND

**Keywords:** kap survey, hcws, vaccination, prevention, infection, hepatitis b

## Abstract

Background: Health Care workers (HCWs) have an approximate four-fold increased risk of contracting hepatitis B infection than the normal population. A lack of knowledge and practices regarding precautions has been frequently observed. We aimed to do a knowledge, attitude, and practices (KAP) study regarding hepatitis B prevention measures among HCWs.

Methods: The study enrolled 250 HCWs. A questionnaire-based proforma regarding KAP towards hepatitis B, its causation, and prevention was filled out by each participant.

Results: Mean age (SD) of participants was 31.8 ± 9.1 years with 83 males and 167 females. Subjects were divided into two groups: Group I (House Surgeons and Residents) and Group II (Nursing staff, Laboratory Technicians, Operation Theatre Assistants). All Group I and 148 (96.7%) of Group II subjects had adequate knowledge regarding the professional risk of hepatitis B virus transmission. Knowledge regarding different modes of transmission was less in Group II subjects (Blood (96.1%), Sex (84.3%), percutaneous route (85.6%), and During Birth (83%)) as compared to Group I (100% in all). All subjects in Group I and 134 (90.9%) Group II subjects knew about vaccination as a preventive measure. There was a slight discordance between attitude and practices towards universal precautionary measures in Group II subjects (Use of gloves 96.1% vs 94.8%; Safe needle disposal 96.7% vs 96.1%; Vaccination 94.8% vs 67.9%). Of the subjects in Group I, 94.8% were vaccinated and 67.9% were vaccinated in Group II, with complete vaccination rates being 76.3% and 43.1%, respectively, and the difference was statistically significant (P < 0.001).

Conclusion: Better knowledge and attitude led to more adoption of preventive practices. But, still, there is a gap in the KAP towards hepatitis B preventive practices and knowledge is not getting transformed into practices. We recommend that all HCWs should be questioned about their vaccination status. In addition, vaccination coverage, various preventive campaigns, and the hospital infection control committee (HICC) need to be strengthened.

## Introduction

Hepatitis B virus (HBV) infection is a major public health problem and is among the leading causes of cirrhosis of liver and hepatocellular carcinoma (HCC). According to World Health Organization (WHO), an estimated 257 million people are living with chronic hepatitis B (CHB) infection with approximately 887,000 deaths occurring annually due to it [1]. India harbours 10-15% of the entire pool of HBV carriers of world with an estimated prevalence of 2-8% among the general population and placing it in intermediate endemic zone [2,3].

Healthcare workers (HCWs) have an approximately four-fold increased risk of acquiring infection as compared to the general population [4,5]. Blood contains the highest HBV titres among all body fluids with risk of transmission varying from 1% to 6% in hepatitis B e antigen (HBeAg) negative cases to 22-31% in HBeAg positive chronic hepatitis B cases [6,7]. Studies have reported low compliance with the practices of universal precautions such as safe needle disposal, wearing gloves during phlebotomy, and using goggles among HCWs [8,9], with injection safety standards ranging from 6% to 22.6% [10]. Despite availability of effective vaccine against HBV infection, vaccination trends in HCWs have been disappointing with a large proportion being still unvaccinated. Moreover, paramedics have a higher risk of HBV transmission than doctors secondary to meagre vaccination and less adoption of safety measures secondary to beliefs, organizational, political and cultural factors [11,12].

Our hospital is one of the largest tertiary care centers in North India and the vaccine is available free of cost to all employees. However, the authors frequently get calls from HCWs regarding needle prick injury and need for post exposure prophylaxis. Various studies across the globe had shown marked diversity among HCWs with regard to knowledge, attitude, and practices (KAP) towards HBV infection, its prevention, and vaccination. Taking these facts into consideration, we aimed to do a KAP study as there is an unmet need to fill this KAP gap in HCWs.

## Materials and methods

**Figure 1:**
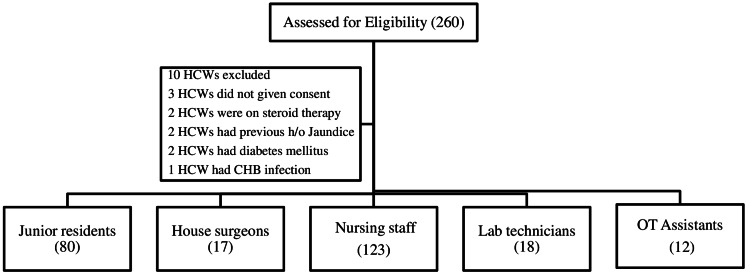
Diagram showing HCWs enrollment in the study. HCWs: Health Care Workers, CHB: Chronic Hepatitis B, OT: Operation Theatre

The study was conducted among HCWs of different departments at Pandit Bhagwat Dayal Sharma Post Graduate Institute of Medical Sciences, Rohtak, Haryana, India, from March 2019 to January 2020. A total of 260 HCWs were screened for this study. HCWs with diabetes, HIV, cirrhosis, previously known chronic HBV/hepatitis C virus (HCV) infection, and on immunosuppressants/chemotherapeutic agents were excluded from the study. Ten subjects were excluded (three HCWs did not give consent, two HCWs were on steroid therapy, two HCWs had a previous history of jaundice, two HCWs had diabetes mellitus, and one HCW had chronic hepatitis B (CHB) infection). Finally, a total of 250 HCWs were included in the study (Figure 1).

The study participants were preferentially from those departments where they were in direct contact with patients and clinical materials with a potentially high risk of horizontal transmission. Out of 250 HCWs, 80 junior residents, 17 house surgeons, 123 nursing staff, 18 lab technicians ( LTs), and 12 operation theatre assistants (OTAs) were taken into the study according to probability proportion to size. A sampling frame (N) of all high-risk HCWs providing care to patients, proportionate to size allocation, was applied to determine the proportion of each category of HCWs. This was multiplied by the sample size to ascertain the actual number of HCWs per cadre (n) as depicted in Table 1.

**Table 1:** Sampling of HCWs by probability proportional to size HCWs: Health Care Workers; OT: Operation Theatre

S. No.	Category of HCW	Number in health facility (N)	Calculated proportion	Number allocated to study (n)
1.	Junior Residents	708	708/2208×250=80	80
2.	House surgeons	150	150/2208×250=17	17
3.	Nursing staff	1088	1088/2208×250=123	123
4.	Lab technicians/ OT technicians	262	262//2208×250=30	30
5.	Total	2208		250

After explaining the objectives of the study, written informed consent was taken from each participant. For data interpretation, subjects were divided into two groups: Group I including medical workers (House Surgeons and Residents) and Group II including para-medical workers (Nursing staff, LTs, OTAs). Blood samples were taken from the participants for HBsAg estimation and the samples were processed by enzyme-linked immunoassay (ELISA) method. We formulated a questionnaire in a structured designed proforma regarding KAP towards HBV, its causation, and prevention. All HCWs were asked to fill it out. The study was approved by the Institutional Ethics Committee of Pandit Bhagwat Dayal Sharma Post Graduate Institute of Medical Sciences (approval no: Ped/18/3104-13/ dated October 20, 2018). 

Statistical analysis

The data was entered in a predesigned proforma. Computer files were created in Microsoft Excel for Windows (Microsoft Corporation, Redmond, Washington, United States). Data analysis was done using Stata Statistical Software: Release 11 (2009; StataCorp LP, College Station, Texas, United States) and SPSS Statistics for Windows, Version 17.0 (Released 2008; SPSS Inc., Chicago, United States). The normally distributed variables were expressed as mean ± standard deviation (SD), and continuous variables with skewed distribution as median (range). Categorical data were presented as proportions and analyzed using chi-square test and Fisher exact test as appropriate.

## Results

Demographic profile

Among participants, 83 (33.2%) were males and 167 (66.8%) were females. The mean age (SD) of participants was 31.8 ± 9.1 years. Eighty (32%) junior residents and 17 (6.8%) house surgeons were taken in Group I (97; 38.8%) whereas 123 (49.2%) staff nurses, 18 (7.2%) LTs, and 12 (4.8%) OTAs formed Group II (153; 61.2%). Nursing staff and junior residents formed the majority of participants accounting for 123 (49.2%) and 80 (32%) subjects, respectively, as shown in Table 2. 

**Table 2:** Demographic and occupational characteristics of health care workers (n =250) OT: Operation Theatre

Variables	No of subjects (n=250)
Age (mean ± SD) (in years)	31.8 ± 9.1
Sex (Male: Female)	83 :167 (1:2)
Group I	97 (38.8%)
Junior Residents	80 (32)
House Surgeons	17 (6.8)
Group II	153 (61.2%)
Nursing Staff	123 (49.2)
Lab Technicians	18 (7.2)
OT Assistants	12 (4.8)

Knowledge of HCWs on HBV transmission

All Group I and 148 (96.7%) of Group II subjects agreed that they are at risk of contracting HBV infection due to their profession. Group I subjects had better knowledge regarding all modes of transmission as compared to Group II (Table 3) with a statistically significant difference. A total of 147 (96.1%) subjects in Group II knew about blood products as a potential source of infection but transmission through sex, percutaneous route, and mother-to-child transmission was known to only 129 (84.3%), 131 (85.6%), and 127 (83%) subjects, respectively. Furthermore, HBV as a cause of liver cancer was known to 94 (96.9%) and 89 (58.2%) subjects in groups I and II, respectively.

**Table 3:** Knowledge of HCWs on hepatitis B virus transmission and its prevention HCWs: Health Care Workers

	Group I (97) n (%)	Group II (153) n (%)	P value
Mode of transmission			
Blood	97 (100)	147 (96.1)	0.084
Sex	97 (100)	129 (84.3)	<0.0001
Percutaneous	97 (100)	131 (85.6)	<0.0001
During birth	97 (100)	127 (83)	<0.0001
Profession risk for hepatitis B virus transmission	97 (100)	148 (96.7)	0.160
Hepatitis B virus as a cause for liver cancer	94 (96.9)	89 (58.2)	<0.0001

Knowledge of HCWs on preventive practices toward occupation risk

All Group I subjects knew that HBV infection is preventable and were aware of the vaccination and other safe practices (wearing gloves, safe needle disposal) as precautionary measures. On the contrary, 149 (97.4%) Group II subjects stated it to be a preventable disease. Regarding safety measures, wearing gloves and safe needle disposal were known to 149 (97.4%) and 147 (96.1%) Group II patients as compared to 100 % in Group I. Only 139 (90.9%) subjects in Group II knew that vaccination can prevent infection and the difference was statistically significant as compared to Group I (P <0.001) as shown in Table 4.

**Table 4:** Knowledge of HCWs on preventive practices toward occupation risk HCWs: Health Care Workers

	Group I (97)	Group II (153)	P Value
Is hepatitis B preventable	97 (100)	149 (97.4)	0.180
How HBV infection can be prevented			
Wearing Gloves	97 (100)	149 (97.4)	0.180
Safe Needle Disposal	97 (100)	147 (96.1)	0.08
Vaccination	97 (100)	139 (90.9)	<0.001

Attitude and practices towards prevention among HCWs

Regarding attitude towards preventive practices, all Group I workers agreed that they should use gloves during various procedures and vaccination should be compulsory for all HCWs. On the other hand, 147 (96.1%) subjects of Group II agreed on the use of gloves during various procedures with only 145 (94.8%) subjects stating the need for compulsory vaccination for all HCWs. All Group I and 96.7% of Group II subjects agreed to the adoption of safe needle disposal. All HCWs in Group I used gloves during various procedures and practiced safe needle disposal whereas 145 (94.8%) and 147 (96.1%) in Group II wore gloves and practiced safe needle disposal, respectively (Table 5). In addition, 92 (94.8%) HCWs in Group I had received vaccination with 74 (76.3%) having complete vaccination. On the other hand, 104 (67.9%) HCWs in Group II had received vaccination with complete vaccination reported in 66 (43.1%). The difference between the two groups with respect to vaccination and complete vaccination was statistically significant (P<0.001).

**Table 5:** Attitude and practices towards prevention among HCWs HCWs: Health Care Workers

Attitude	Group I (n=97)	Group II (n=153)	p-value
Should you use gloves during various procedures	97 (100)	147 (96.1)	0.08
Should you practice safe needle disposal protocol	97 (100)	148(96.7)	0.08
Should vaccintion be compulsory for all HCWs	97 (100)	145 (94.8)	0.025
Practices			
Do you use gloves during procedures	97 (100)	145 (94.8%)	0.025
Do you practice safe needle disposal protocol	97 (100)	147 (96.1)	0.08
Vaccination done	92 (94.8%)	104 (67.9%)	<0.001
Complete vaccination done	74 (76.3%)	66 (43.1%)	<0.001

## Discussion

Occupational exposure to body fluids has been a well-recognized risk factor for HBV infection among HCWs with higher prevalence of HBV in HCWs as compared to the normal population [13]. We conducted this KAP study among 250 HCWs. Number of nursing staff was the highest (49.2%) among the subjects. Since females predominate as the nursing staff almost everywhere in the country so we had almost twice the females (66.8%) as compared to males (33.2%).

Group I subjects had adequate knowledge regarding the professional risk of HBV transmission and various modes of transmission as compared to Group II subjects (Table 3). These results were better than that reported by other studies regarding knowledge of professional risk [14-17] and route of transmission among subjects [15-17,18]. In Group II subjects, 96.7 % stated that they are at risk of contracting HBV infection due to their profession; however, only 85.6% of subjects knew the percutaneous route as a transmission mode. This seems to be a matter of concern as the percutaneous route has been shown to be the most common cause of HBV infection among HCWs in various studies [19-22]. Since education trains individuals to acquire, evaluate, and use information [23], our findings also highlighted the same and revealed an association between the HCW category, level of education, and knowledge of the professional risk of HBV transmission.

All Group I and 97.4% of Group II subjects agreed that HBV infection is preventable. Regarding the prevention measures, there were differences in knowledge in groups I and II subjects, further affirming the association between the educational level of a person and knowledge of occupational hazards. Analyzing the attitude and practices of HCWs towards prevention, all Group I and the majority of Group II workers agreed that they should wear gloves and do safe needle disposal protocol with a slight discordance regarding practices in Group II subjects (Table 5). In addition, all Group I and 94.8 % of Group II subjects stated that vaccination should be compulsory for all HCWs. However, 94.8% of HCWs in Group I and 67.9% in Group II had received vaccination at any point in time with complete vaccination rates being further disappointing with only 76.3% and 43.1% in groups I and II subjects, respectively. The good association between attitude and practices towards wearing gloves and safe needle disposal practices vs dismal association in vaccination trends may be due to the nature of our study. Since our study was questionnaire-based and not directly observed, the position on the real practices carried out by HCWs can’t be commented on. Though our results regarding vaccination were better (94.8% in Group I and 67.9% in Group II) than that reported earlier by Singhal et al. (56.5%) [24] and Sukriti et al. (55.4%) [25], yet these trends clearly indicate that the knowledge and attitude towards preventive measures are not getting transformed into practices necessitating a major concern.

Recommendations

The authors suggest the screening of all HCWs for HBV. A central database has to be secured at the workplace with regular upgradation of the vaccination status of HCWs. The HCWs should be given timely reminders on the vaccination schedule and the defaulters must be notified. In addition, the hospital infection control committee (HICC) needs to be strengthened and regular guidance for waste disposal to all HCWs must be ensured. The strengthening of vaccination coverage, didactic lectures on HBV infection spread, and various prevention campaigns at institute, district, and state levels might be of help to boon the existing system.

Limitations

The study has some inherent limitations. Data on KAP was self-­reported and could be subjected to individual bias. We did not estimate the anti-hepatitis B surface antigen (HBsAb), which might have reflected the true estimates of immunogenicity towards hepatitis B infection in subjects. 

## Conclusions

The study showed the KAP gap in HCWs regarding hepatitis B, and more so in paramedical workers. This gap needs to be filled with the creation of a central database, regular reminders for vaccination, strengthening of the HICC, and waste disposal guidance to all HCWs. We stress that vaccination coverage and various prevention campaigns need more emphasis and strengthening.
